# Embryonal Rhabdomyosarcoma of the Uterine Cervix in a Patient With Xeroderma Pigmentosum: An Exceptional Association

**DOI:** 10.7759/cureus.88544

**Published:** 2025-07-22

**Authors:** Ikram El Motae, Othmane Zouiten, Leila Afani, Mohamed El Fadli, Rhizlane Belbaraka

**Affiliations:** 1 Department of Medical Oncology, Mohammed VI University Hospital of Marrakech, Marrakech, MAR

**Keywords:** consanguinity, embryonal rhabdomyosarcoma, genetic disorder, rare tumor, soft tissue sarcoma, uterine cervix, xeroderma pigmentosum

## Abstract

Cervical rhabdomyosarcoma (RMS) is an uncommon malignant tumor. Its presentation in patients with xeroderma pigmentosum (XP), a genetic condition with marked ultraviolet (UV) sensitivity and skin cancer risk, is very rare. We report the case of a teenage girl with XP diagnosed with embryonal rhabdomyosarcoma of the uterine cervix.

A 17-year-old girl with a longstanding history of XP presented with pelvic pain, abnormal vaginal bleeding, dysuria, and constipation. Clinical examination revealed a 7 × 6 cm exophytic cervical mass. Histopathological analysis confirmed the diagnosis of embryonal rhabdomyosarcoma of the cervix. Management included neoadjuvant chemotherapy based on the IVA protocol (ifosfamide, vincristine, and actinomycin D), followed by total hysterectomy and bilateral pelvic lymphadenectomy. The patient is currently undergoing adjuvant chemotherapy, with no clinical or radiological evidence of recurrence.

This exceptional association between XP and cervical embryonal rhabdomyosarcoma presents complex diagnostic and therapeutic challenges. It underscores the importance of multidisciplinary management and heightened vigilance for rare tumors in the context of XP. Further research is warranted to better understand the management of extracutaneous malignancies in patients with XP.

## Introduction

Rhabdomyosarcoma (RMS) is a soft tissue sarcoma of mesenchymal origin. It is the most common sarcoma in children, accounting for approximately 5% of all pediatric malignancies, but represents less than 5% of malignancies in adults. The estimated incidence is 4.5 cases per million per year in individuals aged 1-20 years [1]. The genitourinary tract is the second most frequently affected site after the head and neck region [2]. Among genitourinary RMSs, the vagina is most often involved, whereas cervical involvement is rare, representing only 0.5% of cases [3].

Xeroderma pigmentosum (XP) is a rare autosomal recessive genetic disorder characterized by a defect in nucleotide excision repair (NER), particularly affecting cells exposed to ultraviolet (UV) radiation. With the exception of the XP-V subtype, xeroderma pigmentosum results from mutations in genes encoding proteins of the nucleotide excision repair (NER) system. These mutations lead to the dysfunction of one or more key proteins involved in the recognition and repair of ultraviolet-induced DNA damage. The genes implicated include *XPA*, *XPB* (*ERCC3*), *XPC*, *XPD* (*ERCC2*), *XPE* (*DDB2*), *XPF* (*ERCC4*), and *XPG* (*ERCC5*). In contrast, the XP-V subtype is caused by mutations in the *POLH* gene, which encodes DNA polymerase eta, a specialized enzyme involved in translesion DNA synthesis. This leads to the accumulation of mutations and a markedly increased cancer risk, particularly skin cancers, but also certain internal malignancies [4].

Herein, we describe the case of a 17-year-old girl with a diagnosis of XP since infancy who presented with embryonal rhabdomyosarcoma of the uterine cervix. To our knowledge, this is the first documented case of such an association in the literature.

## Case presentation

**Figure 1:**
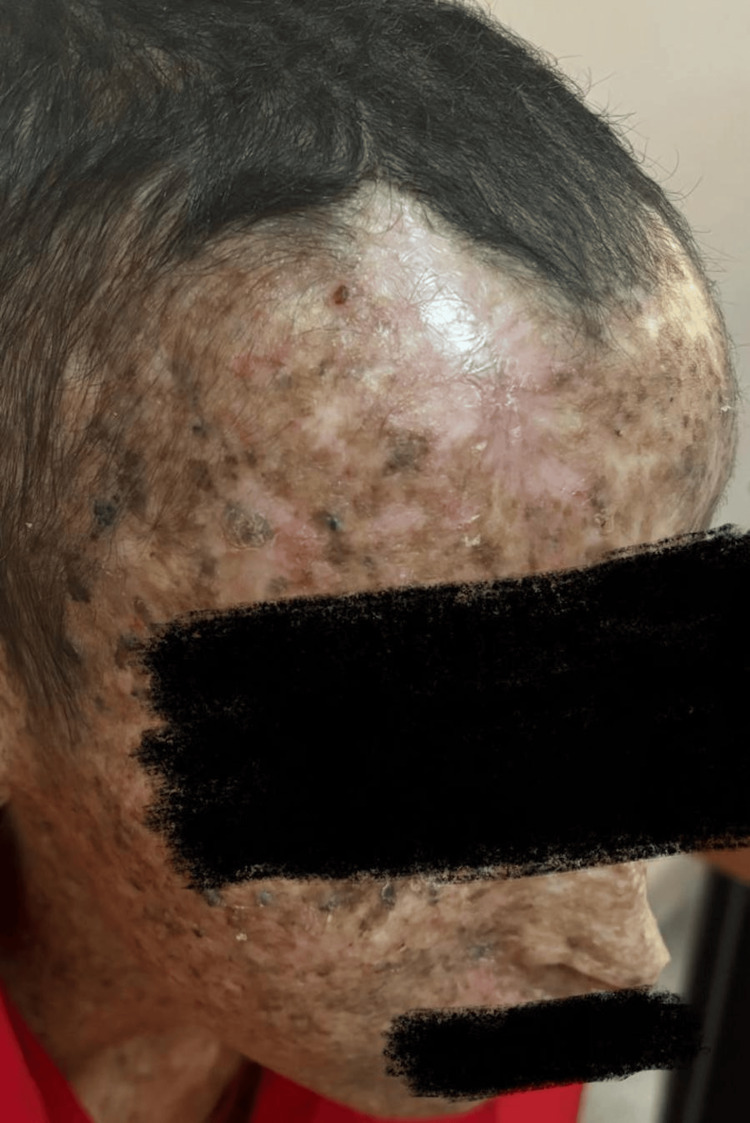
Cutaneous lesions characteristic of xeroderma pigmentosum

A 17-year-old girl born to consanguineous first-degree relatives had been followed by dermatology since the age of six months for XP, diagnosed based on characteristic pigmentary changes in sun-exposed areas (Figure 1). There was no prenatal diagnosis, although an older sister was also affected. No similar symptoms were reported in the parents.

She presented with chronic pelvic pain described as a sensation of heaviness, along with abnormal uterine bleeding, dysuria, and constipation, persisting for several months. On examination, she was hemodynamically and respiratory stable.

**Figure 2:**
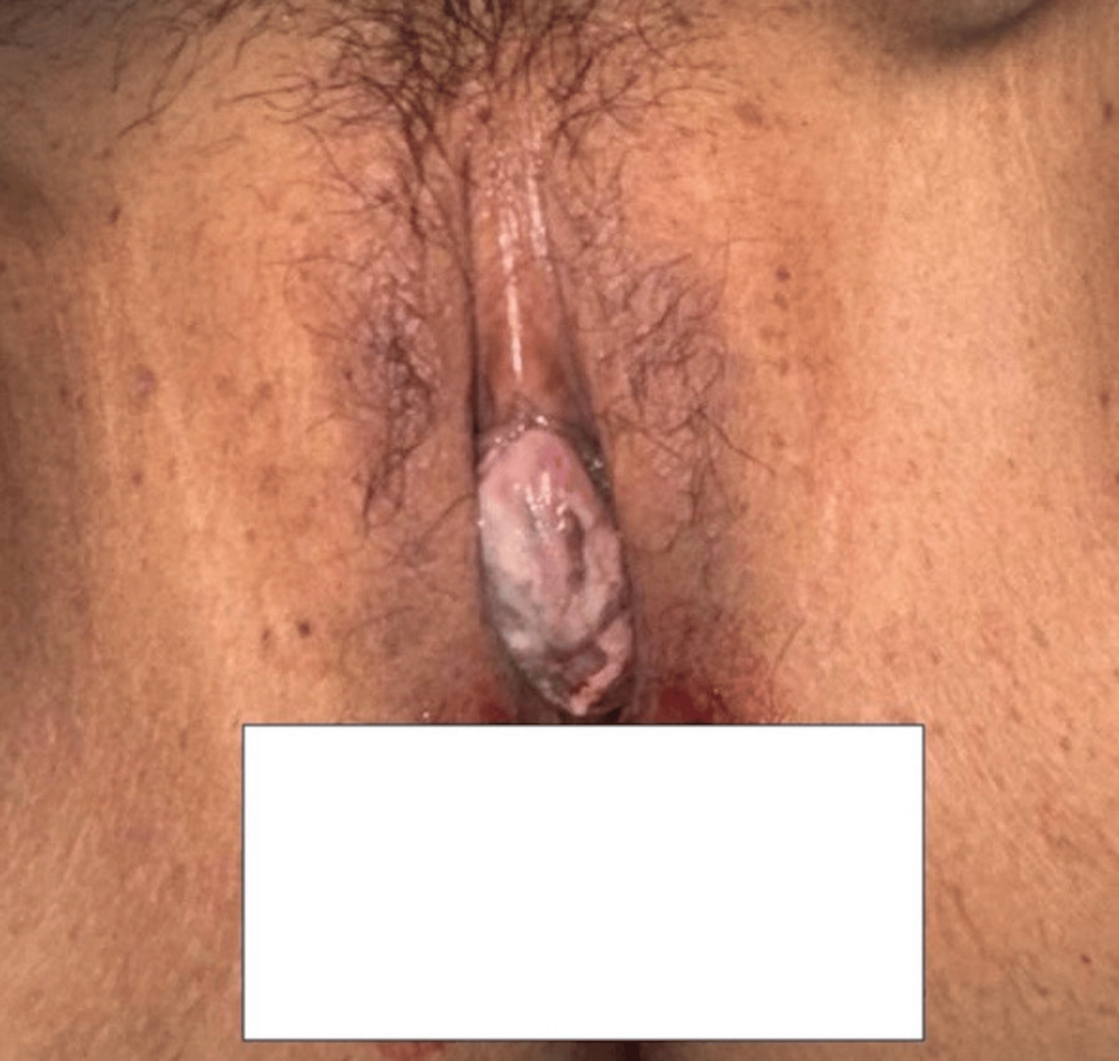
Exophytic friable cervical mass protruding through the vaginal introitus

Gynecological examination revealed a friable, exophytic mass protruding through the vaginal introitus, measuring approximately 7 × 6 cm (Figure 2). Laboratory workup showed microcytic anemia (hemoglobin (Hb): 8.8 g/dL, mean corpuscular volume (MCV): 74.5 fL), with preserved renal and hepatic function (Table 1).

**Table 1:** Summary of key laboratory findings with corresponding reference ranges

Parameter	Result	Reference range
Hemoglobin	8.8 g/dL	12-16 g/dL
Mean corpuscular volume	74.5 fL	78-98 fL
White blood cell count	6,500/mm^3^	4,000-11,000/mm^3^
Platelet count	220,000/mm^3^	150,000-400,000/mm^3^
C-reactive protein	3 mg/L	<5 mg/L
Urea	0.3 g/L	0.15-0.4 g/L
Creatinine	6.2 mg/L	5-9 mg/L
Aspartate aminotransferase	16 IU/L	<40 IU/L
Alanine aminotransferase	20 IU/L	<40 IU/L
Total bilirubin	2.9 mg/L	<10 mg/L

**Figure 3:**
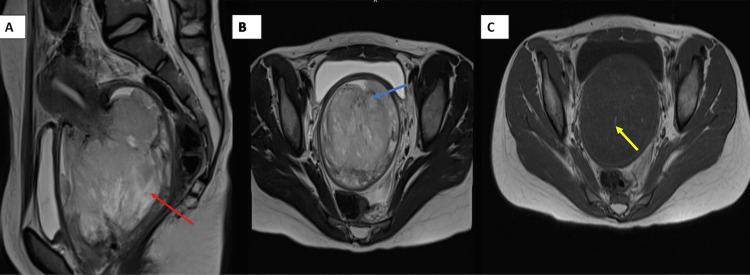
Large exophytic mass occupying the vaginal lumen, arising from the cervix, measuring approximately 86 × 73 mm (axial plane) (A) and 104 mm (sagittal plane) (B), showing heterogeneous high signal intensity on T2-weighted images (A and B) and low signal intensity on T1-weighted images with signs of hemorrhage (C) (A) Axial T2-weighted MRI showing a large exophytic mass arising from the cervix and occupying the vaginal lumen, measuring approximately 86 × 73 mm. The mass displays heterogeneous high signal intensity (red arrow). (B) Sagittal T2-weighted MRI revealing the same mass measuring 104 mm in craniocaudal length, with similar high T2 signal (blue arrow). (C) Axial T1-weighted image demonstrating low signal intensity within the lesion, with focal areas of high signal suggestive of hemorrhage (yellow arrow). MRI: magnetic resonance imaging

Pelvic magnetic resonance imaging (MRI) revealed a large heterogeneous mass with high T2 and low T1 signal intensities, arising from the cervix and filling the vaginal cavity. The mass measured 86 × 73 × 104 mm and filled the cervical os (Figure 3).

Biopsy under anesthesia revealed an undifferentiated, necrotic tumor proliferation. Immunohistochemistry (IHC) showed positivity for desmin and myogenin, confirming embryonal rhabdomyosarcoma of the cervix per the 2020 World Health Organization (WHO) classification.

**Figure 4:**
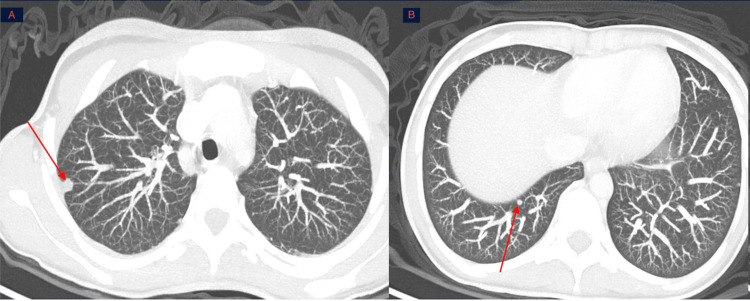
Thoracic CT scan in lung window, axial slices with MIP reconstructions, showing pulmonary nodules and micronodules, the largest measuring 7.3 × 6 mm (A) and 4.4 × 3 mm Thoracic CT scan in lung window (axial slices with MIP reconstructions) demonstrates a dense pulmonary micronodule in the dorsal segment of the right upper lobe, measuring 7.3 × 6 mm (red arrow, A), as well as another micronodule in the right posterobasal segment, measuring 4.4 × 3 mm (red arrow, B). CT: computed tomography, MIP: maximum intensity projection

Thoraco-abdominopelvic computed tomography (CT) scan showed pulmonary micronodules with irregular margins, the largest measuring 7.3 × 6 mm and 4.4 × 3 mm, suggestive of pulmonary metastases (Figure 4).

The case was discussed at a multidisciplinary tumor board. Neoadjuvant chemotherapy with the IVA protocol (ifosfamide, vincristine, and actinomycin D) was initiated. After four cycles, the patient showed significant clinical improvement (resolution of pain, abnormal bleeding, and compressive symptoms) and partial radiological response (RECIST 1.1), with reduced tumor size and stable pulmonary nodules.

A total hysterectomy with adnexa preservation and bilateral iliac lymphadenectomy was performed. Histopathology confirmed cervical, isthmic, and uterine body infiltration by malignant small round cells consistent with embryonal RMS.

The patient is currently receiving postoperative chemotherapy according to the IVA protocol, which is well tolerated both clinically and biologically, with no evidence of local recurrence.

## Discussion

Xeroderma pigmentosum (XP) is a rare genetic disorder with autosomal recessive inheritance. Its prevalence is estimated at one in 300,000 in Europe and the United States, but it is significantly higher in regions with a high rate of consanguinity, particularly in North Africa. The geographic distribution of the different genetic groups is heterogeneous, with certain forms predominating in specific regions: group C is most frequently reported in Mediterranean countries, while groups A and F are more common in Japan. Affected patients have a markedly increased risk of skin cancers (up to 100 times higher than in the general population), as well as a 10- to 20-fold increased risk of developing extracutaneous tumors such as leukemias, thyroid cancers, uterine sarcomas, and brain or lung tumors [4].

The mechanisms explaining the occurrence of tumors in internal organs not exposed to UV radiation in patients with XP remain poorly understood. In certain subtypes, such as XP-C, DNA alterations have been observed in cells of the central nervous system despite the absence of UV exposure [5]. This genetic deficiency leads to increased susceptibility to DNA damage in various tissues, which could explain the occurrence of neuropathies and extracutaneous cancers [5]. Other factors, such as oxidative stress or tobacco exposure, may also exacerbate this damage. For example, a meta-analysis by Dou et al. demonstrated a higher incidence of bladder cancer in the XP-C subtype, linked to a DNA repair defect aggravated by tobacco smoke exposure [5]. This mechanism could thus explain the occurrence of non-cutaneous tumors, such as the rhabdomyosarcoma observed in our patient. Although cases of gynecological cancers have been reported in patients with XP, no cervical rhabdomyosarcoma has yet been described in this context. We will now discuss the particularities of this rare localization.

Rhabdomyosarcoma (RMS) is the most common form of soft tissue sarcoma in children, accounting for more than 50% of pediatric cases [1]. The most recent classification by the World Health Organization in 2020 defines four subtypes: embryonal rhabdomyosarcoma, alveolar rhabdomyosarcoma, pleomorphic rhabdomyosarcoma, and spindle cell/sclerosing rhabdomyosarcoma. The embryonal subtype is common in children and adolescents [6]. Embryonal RMS of the uterine cervix most often presents with vaginal bleeding and an exophytic or polypoid cervical mass, which can sometimes protrude through the introitus [7]. MRI is the most commonly used imaging modality, with an approximate tumor detection rate of 83% [7]. Lymph node involvement and metastatic spread remain rare. When they occur, metastases mainly affect the lungs [7,8], which is consistent with our observation. Immunohistochemistry (IHC) aids in the diagnosis of RMS. Commonly used markers include antibodies against desmin, muscle-specific actin, and myogenin. Vimentin is generally positive but is not specific to RMS [7].

The treatment of RMS is based on a multimodal approach, including surgery, chemotherapy, and radiotherapy. Treatment is challenging because it involves young women who wish to preserve their fertility [8]. Chemotherapy has been an integral part of RMS treatment since the 1960s. The VAC protocol (vincristine, actinomycin D, and cyclophosphamide) is the standard in North American countries. In Europe, the IVA protocol (ifosfamide, vincristine, and actinomycin D) has demonstrated similar results [9]. Radiotherapy is the third treatment modality and is indicated for local tumor control in cases of microscopic or macroscopic residual disease following surgery or chemotherapy [8].

The prognosis of RMS depends on age, location, histological type, and stage. Compared with children, adults have a poorer prognosis. According to a comparative study involving 2,600 patients, the five-year overall survival rate was 27% in adults versus 61% in children [10].

## Conclusions

This case highlights a rare association between xeroderma pigmentosum and embryonal rhabdomyosarcoma of the uterine cervix, expanding the spectrum of tumors related to XP. It underscores the need for a cautious, individualized multimodal management approach due to the underlying genetic fragility and increased risk of toxicity. Close oncological monitoring and multidisciplinary consultation are essential to optimize follow-up and therapeutic decisions. This rare case encourages further research into tumorigenic mechanisms in the context of XP.
